# Wnt/β-catenin signaling mediates the abnormal osteogenic and adipogenic capabilities of bone marrow mesenchymal stem cells from chronic graft-versus-host disease patients

**DOI:** 10.1038/s41419-021-03570-6

**Published:** 2021-03-23

**Authors:** Han-zhou Qi, Yi-ling Ye, Yuan Suo, Hong Qu, Hai-yan Zhang, Kai-bo Yang, Zhi-ping Fan, Fen Huang, Li Xuan, Yan-qiu Chen, Hua Jin, Qi-fa Liu

**Affiliations:** Department of Hematology, Nanfang Hospital, Southern Medical University, Guangzhou, China

**Keywords:** Cell signalling, Mesenchymal stem cells

## Abstract

Chronic graft-versus-host disease (cGVHD) is the main cause of non-relapse mortality after allogeneic hematopoietic stem cell transplantation (allo-HSCT). Mesenchymal stem cells (MSCs) in bone marrow (BM) remain unclear in the pathophysiology of cGVHD. In this study, we analyzed BM-MSCs from 66 patients after allo-HSCT, including 33 with active cGVHD and 33 without cGVHD. BM-MSCs showed similar morphology, frequency, phenotype, and proliferation in patients with or without cGVHD. MSCs from the active cGVHD group showed a decreased apoptosis rate (*P* < 0.01). Osteogenic capacity was increased while adipogenic capacity was decreased in the active cGVHD MSCs compared with no-cGVHD MSCs. The expressions of osteogenic gene RUNX2 and COL1A1 were higher (*P* < 0.001) while adipogenic gene PPAR-γ and FABP4 were lower (*P* < 0.001) in the active cGVHD MSCs than no-cGVHD MSCs. These changes were associated with the severity of cGVHD (*P* < 0.0001; *r* = 0.534, *r* = 0.476, *r* = −0.796, and *r* = −0.747, respectively in RUNX2, COL1A1, PPAR-γ, and FABP4). The expression of Wnt/β-catenin pathway ligand Wnt3a was increased in cGVHD-MSCs. The dysfunction of cGVHD-MSCs could be reversed by Dickkopf related protein 1(DKK1) to inhibit the binding of Wnt3a. In summary, the differentiation of BM-MSCs was abnormal in active cGVHD, and its underlying mechanism is the upregulated of Wnt3a through Wnt/β-catenin signaling pathway of MSCs.

## Introduction

Allogeneic hematopoietic stem cell transplantation (allo-HSCT) is widely used in the treatment of hematopoietic and non-hematopoietic diseases. Chronic graft-versus-host disease (cGVHD) is a leading cause of non-relapse mortality after allo-HSCT^1–5^. The clinical symptoms of cGVHD are highly variable, including skin sclerosis, bronchiolitis obliterans, as well as salivary and lacrimal glands^6–8^. cGVHD is an autoimmune-like syndrome caused by the interactions of donor CD4^+^ T and B cells and the production of IgG^9,10^. However, the pathophysiology of cGVHD is still not completely understood. Emerging evidence from mice and human studies has demonstrated that abnormalities in the bone marrow niche play an important role in the pathogenesis of cGVHD^11,12^. Mesenchymal stem cells (MSCs) are a form of multipotent adult stem cells that can be isolated from bone marrow (BM) and other tissues^13–15^. MSCs possess the multipotency and immunomodulatory capabilities, including the capacity to differentiate into a variety of cell types such as osteoblasts, chondrocytes, adipocytes, and myoblasts^16,17^, and the capacity to suppress immunological responses such as the suppression of CD4^+^ T cells, CD8^+^ T cells and B cells proliferation and induction of Tregs^18^. Emerging evidence has demonstrated that MSCs participate in the pathogenesis of autoimmune diseases such as rheumatoid arthritis (RA), systemic lupus erythematosus (SLE), and ankylosing spondylitis (AS)^19–21^. For example, the number of MSCs and the osteogenic capacity increased in the RA mouse model^20^, and MSCs from the patients with SLE had a morphological appearance of senescence and impaired capabilities of differentiation^19^. In regard to the relationship between MSCs and the pathogenesis of GVHD, a few studies reported that the number of MSCs were decreased and differentiation was abnormal in aGVHD patients^22,23^. But it is rarely reported whether MSCs were abnormal in cGVHD patients.

With regard to the underlying mechanism of the abnormality of MSCs in autoimmune diseases, some studies demonstrated that the upregulation of the Wnt/β-catenin pathway was associated with the dysfunction of MSCs^24–26^. In GVHD recipients, the mechanism of MSCs dysfunction is unclear. In the present study, we investigated the dysfunction of MSCs in patients with cGVHD and its underlying mechanism.

## Results

### Patient characteristics

Sixty-six patients were enrolled in this study from January 2017 to February 2019. There were 38 males and 28 females, with a median age of 31 (range: 15–55) years. There were no significant differences in age, gender, primary disease, time post-transplantation, conditioning regimen, transplant type, source of stem cell, GVHD prophylaxis, or grade of acute GVHD between patients with and without cGVHD (Table 1). Among patients with active cGVHD, the median time from HSCT to cGVHD diagnosis was 8 (range: 3.4–20.8) months, and the median time from cGVHD diagnosis to sample collection was 2.2 (range: 0.2–7.7) months. The most frequent organ manifestations of cGVHD were skin (69.7%), liver (45.5%), and oral mucosa (42.4%). Fourteen patients (52.4%) had more than two organs involved. Clinical manifestations of cGVHD are summarized in Table 2.

**Table 1:** Patient characteristics.

	Chronic GVHD		
Characteristic	No (*n* = 33)	Active (*n* = 33)	*P*
Age, median (range), y	26 (15–55)	32 (15–55)	0.40
Gender, *n* (%)			0.08
Male	14 (42.4)	21 (63.6)	
Female	19 (57.6)	12 (36.4)	
Primary disease, no (%)^a^			0.34
AML	24 (72.7)	19 (57.6)	
ALL	5 (15.2)	11 (33.3)	
Others	4 (12.1)	3 (9.1)	
Time from HSCT to sample collection (range), months	12.8 (1.5–24.0)	9.5 (3.6–21.1)	0.11
Conditioning regimen, no (%)^b^			0.14
Myeloablative	16 48.5)	22 (66.7)	
Intensified	17 (51.5)	11 (33.3)	
HLA type, no(%)			0.13
MSD	13 (39.4)	17 (51.5)	
MUD	6 (18.2)	1 (3.0)	
HID	14 (42.4)	15 (45.5)	
Source of stem cell, no (%)			0.13
PBSC	20 (60.6)	19 (57.6)	
BM + PBSC	13 (39.4)	14 (42.4)	
GVHD prophylaxis, no (%)^c^			1.00
ATG based	16	16	
Non-ATG based	17	17	
History of acute GVHD grade, no (%)			0.80
0–1	15 (45.5)	14 (42.4)	
2–4	18 (54.5)	19 (57.6)	
No. of immunosuppressive therapies before inclusion (%)^d^			<0.001
None	20 (60.6)	0 (0.0)	
1	11 (33.3)	4 (12.1)	
2	2 (6.1)	15 (45.5)	
≥3	0 (0.0)	14 (42.4)	

*AML* acute myeloid leukemia, *ALL* acute lymphocytic leukemia, *HSCT* hematopoietic stem cell transplantation, *HLA* human leukocyte antigen, *MSD* HLA-matched sibling donor, *MUD* HLA-matched unrelated donor, *HID* haplo-identical donor, *PBSC* peripheral blood stem cells, *BM* bone marrow, *GVHD* graft-versus-host disease, *ATG* antithymocyte globulin.

^a^MThe other category included mixed lineage acute leukemia, myelodysplastic syndrome, and lymphoma.

^b^Myeloablative conditioning regimens include TBI (total body irradiation) + Cy (cyclophosphamide), Bu (busulfan) + Cy, and Bu + Flu (fludarabine).Intensified conditioning regiments include TBI + Cy + etoposide, and Flu + cytarabine + TBI + Cy.

^c^Non-ATG based GVHD prophylaxis include cyclosporine A (CsA),methotrexate (MTX), and mycophenolate mofetil (MMF). ATG based GVHD prophylaxis include CsA + MTX + MMF + ATG.

^d^Immunosuppressive treatments include CsA, tacrolimus (Tac), MMF, and steroids.

**Table 2:** Clinical manifestations of cGVHD.

Organ	Mild	Moderate	Severe
	*N* = 8	*N* = 11	*N* = 14
Skin (%)	5 (15.2)	5 (15.2)	13 (39.4)
Liver (%)	3 (9.1)	6 (18.2)	6 (18.2)
Gastrointestinal (%)	0 (0.0)	0 (0.0)	3 (9.1)
Oral mucosa (%)	3 (9.1)	4 (12.1)	7 (21.2)
Eye (%)	2 (6.1)	0 (0.0)	2 (6.1)
Lung (%)	0 (0.0)	0 (0.0)	3 (9.1)
Joint (%)	0 (0.0)	1 (3.0)	0 (0.0)
Genital Tract (%)	0 (0.0)	1 (3.0)	0 (0.0)

### Phenotype and frequency of BM-MSCs

**Fig. 1:**
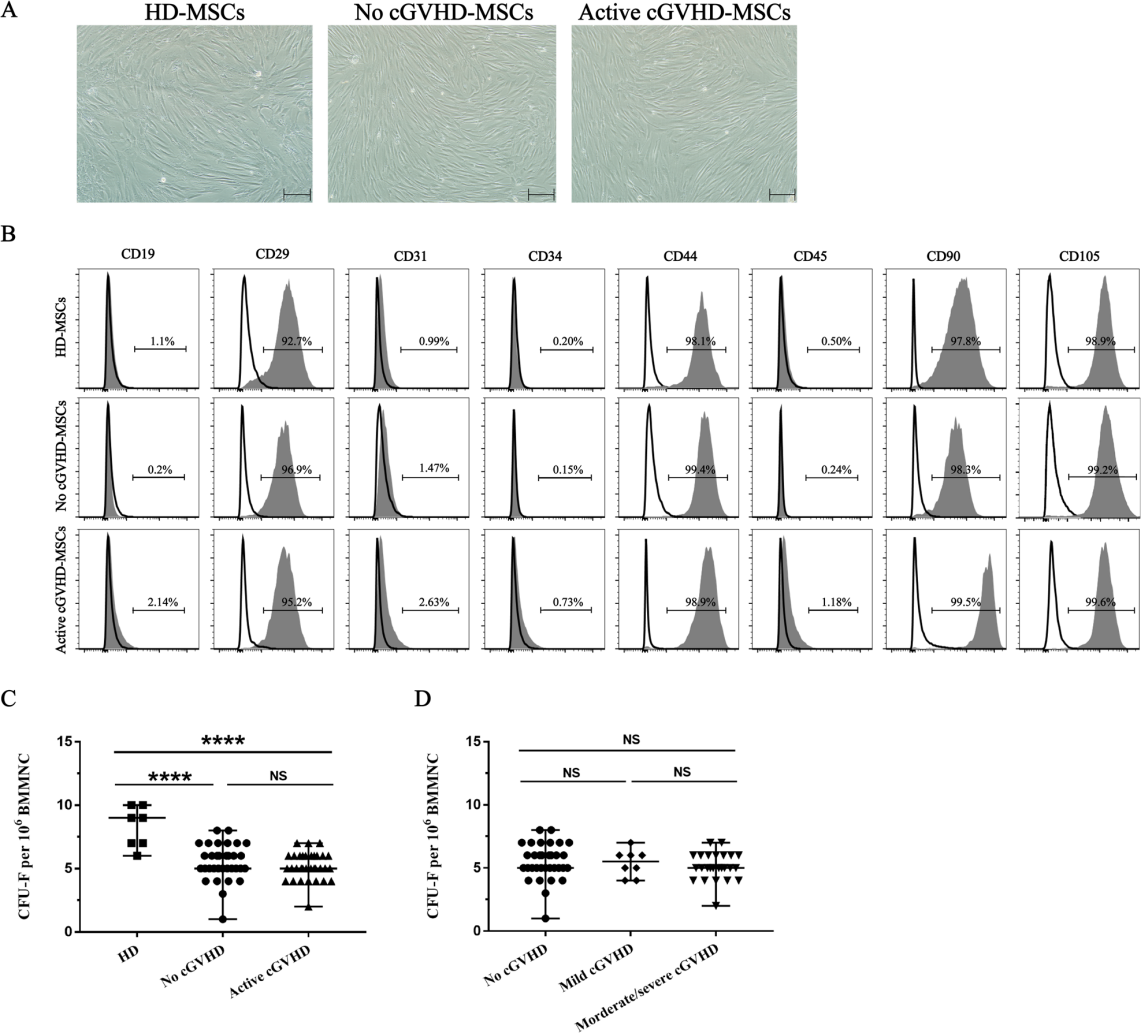
Similar morphology, immunophenotype, and frequency between MSCs from patients with active cGVHD, patients without cGVHD and heathy donors. **A** Representative morphology of HD-MSCs, no cGVHD-MSCs, and active cGVHD-MSCs. Scale bar = 200 µm. **B** Representative flow cytometric characterization of cell surface marker expression on MSCs in healthy donors, patients without cGVHD and patients with active cGVHD. Gray-filled histograms depict the expression of CD29, CD44, CD90, and CD105 and the absence of CD19, CD31, CD34, and CD45. Isotypic controls are represented by the open histograms. **C** Frequency of MSCs in healthy donors, patients without cGVHD, and patients with active cGVHD. **D** Frequency of MSCs in patients with different severities of cGVHD. HD-MSCs (*N* = 7), no cGVHD MSCs (*N* = 33), mild cGVHD-MSCs (*N* = 8) and morerate/severe cGVHD (*N* = 25). Black bars in each figure represent the median ± range. NS, not significant.

MSCs from patients with active cGVHD, no cGVHD, and healthy donors (HD) showed a similar fusiform morphology (Fig. 1A). To investigate the characteristics of the MSCs, flow cytometry was analyzed the cell surface markers, including CD29, CD44, CD90, CD105, CD19, CD31, CD34, and CD45. The results revealed that active cGVHD-MSCs, no cGVHD-MSCs, and HD-MSCs shared the same expression profile (Fig. 1B). The frequencies of active cGVHD-MSCs and no cGVHD-MSCs were significantly lower than those of HD-MSCs (5, 5, and 9, per 10^6^BMMNCs, *P* < 0.001, Fig. 1C). But there were no differences among the frequencies of different severities in active cGVHD-MSCs and no cGVHD-MSCs (5, 5, and 5, per 10^6^BMMNCs, *P* = 0.57, Fig. 1D).

### Proliferation and apoptosis of MSCs

**Fig. 2:**
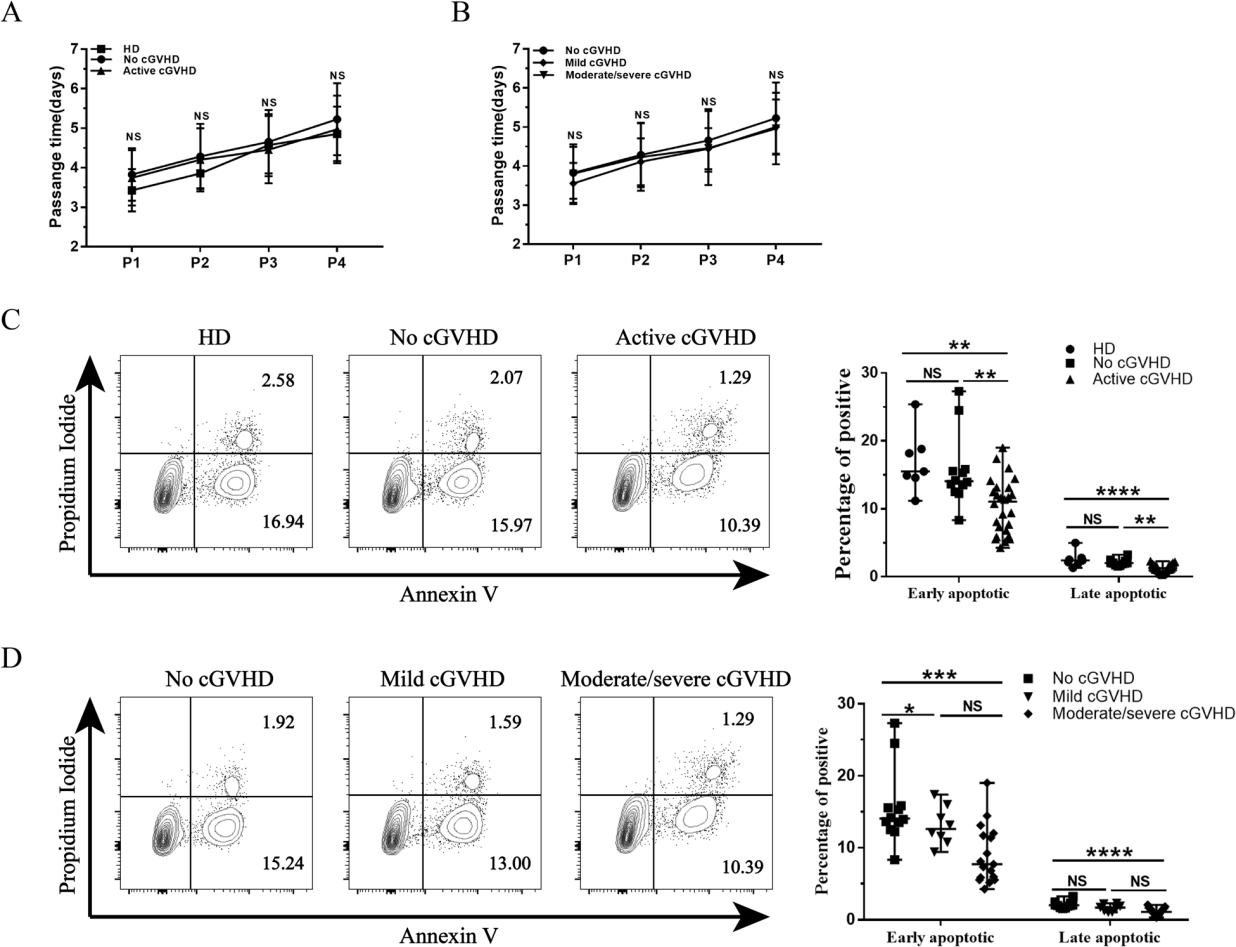
The proliferation of MSCs was similar in the three groups, while apoptotic cells were decreased in MSCs from patients with active cGVHD. **A** Passage time of HD-MSCs (*N* = 7), no cGVHD-MSCs (*N* = 33) and active cGVHD-MSCs (*N* = 33) from P1–P4. **B** Passage time of MSCs from P1–P4 with different severities of cGVHD (*N* = 8 in mild cGVHD and *N* = 25 in morderate/severe cGVHD). **C** Representative apoptosis analysis in MSCs by flow cytometry. The percentage of early and late apoptotic cells (median with interquartile range) in HD-MSCs (*N* = 7), no cGVHD-MSCs (*N* = 12) and active cGVHD-MSCs (*N* = 25) were quantified are shown in histograms. **D** Representative apoptosis analysis in MSCs with different severity of cGVHD (*N* = 8 in mild cGVHD-MSCs and *N* = 17 in moderated/severe cGVHD-MSCs). Black bars in each figure represent the median ± range. NS, not significant. **P < 0.01, ***P < 0.001, and ****P < 0.0001.

There was no difference in proliferation among active cGVHD-MSCs, no GVHD-MSCs, and HD-MSCs by detecting the passage time from P0 to P4(*P* = 0.36, 0.42, 0.60, and 0.37, respectively, Fig. 2A). These similarities were also found between patients with mild cGVHD and patients with moderate/severe cGVHD (P = 0.36, 0.70, 0.96, and 0.91, respectively, Fig. 2B). These findings were also confirmed by CCK-8 cell growth assays (Supplemental Fig. 1). The percentage of early apoptotic MSCs decreased in patients with active cGVHD compared with those of patients without cGVHD (11.08% vs. 14.07%, *P* < 0.01) and those of healthy donors (11.08% vs. 15.50%, *P* < 0.01, Fig. 2C). These decreases were associated with disease severity. Moderate/severe cGVHD-MSCs had a significantly decreased early apoptotic rate compared with mild cGVHD-MSCs (median, 7.71% vs. 13.37%, *P* < 0.05, Fig. 2D). Comparisons of late apoptosis showed a similar trend. Patients with active cGVHD had significantly lower percentage of late apoptotic MSCs than those of patients without cGVHD (median, 1.25% vs. 2.01%, *P* < 0.01) and those of healthy donors (median, 1.25% vs. 2.41%, *P* < 0.0001, Fig. 2C). Further, moderate/severe cGVHD-MSCs had a significantly decreased late apoptotic rate compared with no cGVHD-MSCs (1.09% vs 2.01%, *P* < 0.0001, Fig. 2D).

### Differentiation of MSCs

**Fig. 3:**
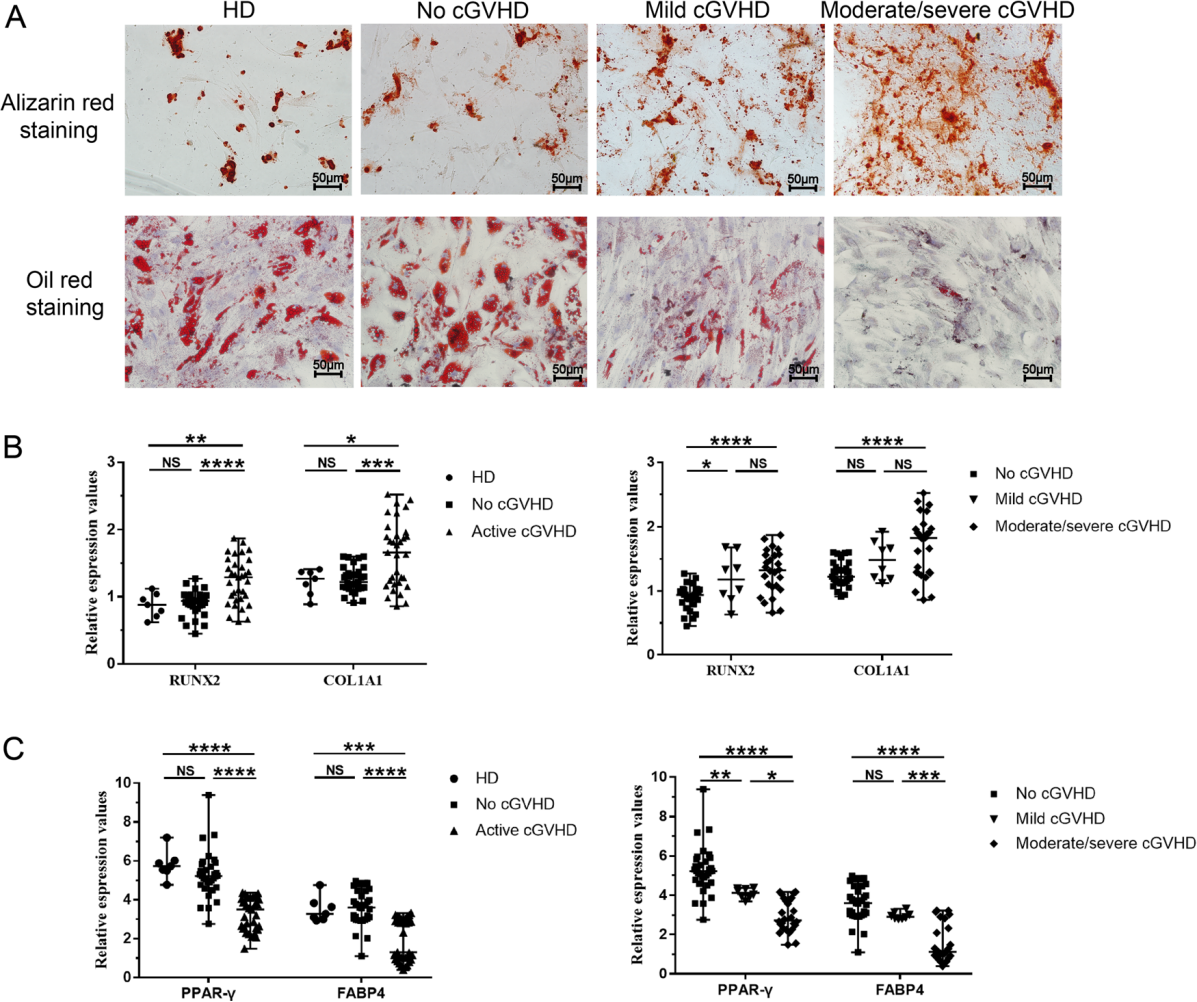
Increased osteogenic differentiation capacity and decreased adipogenic differentiation capacity in MSCs from patients with active cGVHD. **A** Osteogenic differentiation (above) of HD-MSCs, no cGVHD-MSCs, mild cGVHD-MSCs and moderate/severe cGVHD-MSCs was demonstrated by alizarin red staining, Orange-red represents calcium nodules. Adipogenic differentiation (under) of HD-MSCs, no cGVHD-MSCs, mild cGVHD-MSCs, and moderate/severe cGVHD-MSCs was detected by Oil Red O staining, and red represents lipid droplets. Scar bar = 50 μm. **B** Gene expression of osteogenic markers RUNX2 and COL1A1 in HD-MSCs, no cGVHD-MSCs, active cGVHD-MSCs, and different severities of cGVHD-MSCs. **C** Gene expression of adipogenic markers PPAR-γ and FABP4 in HD-MSCs, no cGVHD-MSCs, active cGVHD-MSCs, and different severities of cGVHD-MSCs. HD-MSCs (*n* = 7), no cGVHD-MSCs (*n* = 33), mild cGVHD-MSCs (*n* = 8), and moderate/severe cGVHD-MSCs (*n* = 25). Results were normalized to the expression of glyceraldehyde 3-phosphate dehydrogenase (GAPDH). Black bars in each figure represent the median ± range. NS, not significant. *P < 0.05, **P < 0.01, ***P < 0.001, and ****P < 0.0001.

To investigate whether the differentiation capacity of MSCs in active cGVHD patients, we induced MSCs into osteogenic and adipogenic lineages in vitro. The calcium nodules increased in patients with active cGVHD compared with those of patients without cGVHD and healthy donors. In contrast, the lipid droplets in the active cGVHD group decreased compared with those of no-cGVHD group and HD group (Fig. 3A).

The expression of specific marker genes was further investigated using real-time qPCR. The expressions of the osteogenic markers RUNX2 and COL1A1 were significantly increased in active cGVHD-MSCs compared with no cGVHD-MSCs (1.31 vs 0.94, *P* < 0.001 and 1.66 vs 1.29, *P* < 0.001, respectively) and HD-MSCs (1.31 vs 0.88, *P* = 0.009; and 1.66 vs 1.27, *P* = 0.023, respectively). These increases were more significant in patients with moderate/severe cGVHD than in patients without cGVHD (median, 1.32 vs 0.94, *r* = 0.534, *P* < 0.001; 1.83 vs 1.29, *r* = 0.476, *P* < 0.001, Fig. 3B). In contrast, the expressions of adipogenic markers PPAR-γ and FABP4 were decreased in active cGVHD-MSCs compared with that of no cGVHD-MSCs (3.5 vs 5.21, *P* < 0.001 and 1.48 vs 3.71, *P* < 0.001, respectively) and HD-MSCs (3.5 vs 5.74, *P* < 0.001; and 1.48 vs 3.28, *P* < 0.001, respectively). Moreover, these decreases were associated with cGVHD severity. Moderate/severe cGVHD-MSCs had lower expression of PPAR-γ and FABP4 compared to mild cGVHD-MSCs (2.73 vs 4.03, *P* = 0.017; and 1.13 vs 2.95, *P* < 0.001, respectively) and no cGVHD-MSCs (median, 2.73 vs 5.21, *P* < 0.001; and 1.13 vs 3.71, *P* < 0.001, respectively, Fig. 3C).

### Upregulation of the Wnt/β-catenin pathway in active cGVHD-MSCs

**Fig. 4:**
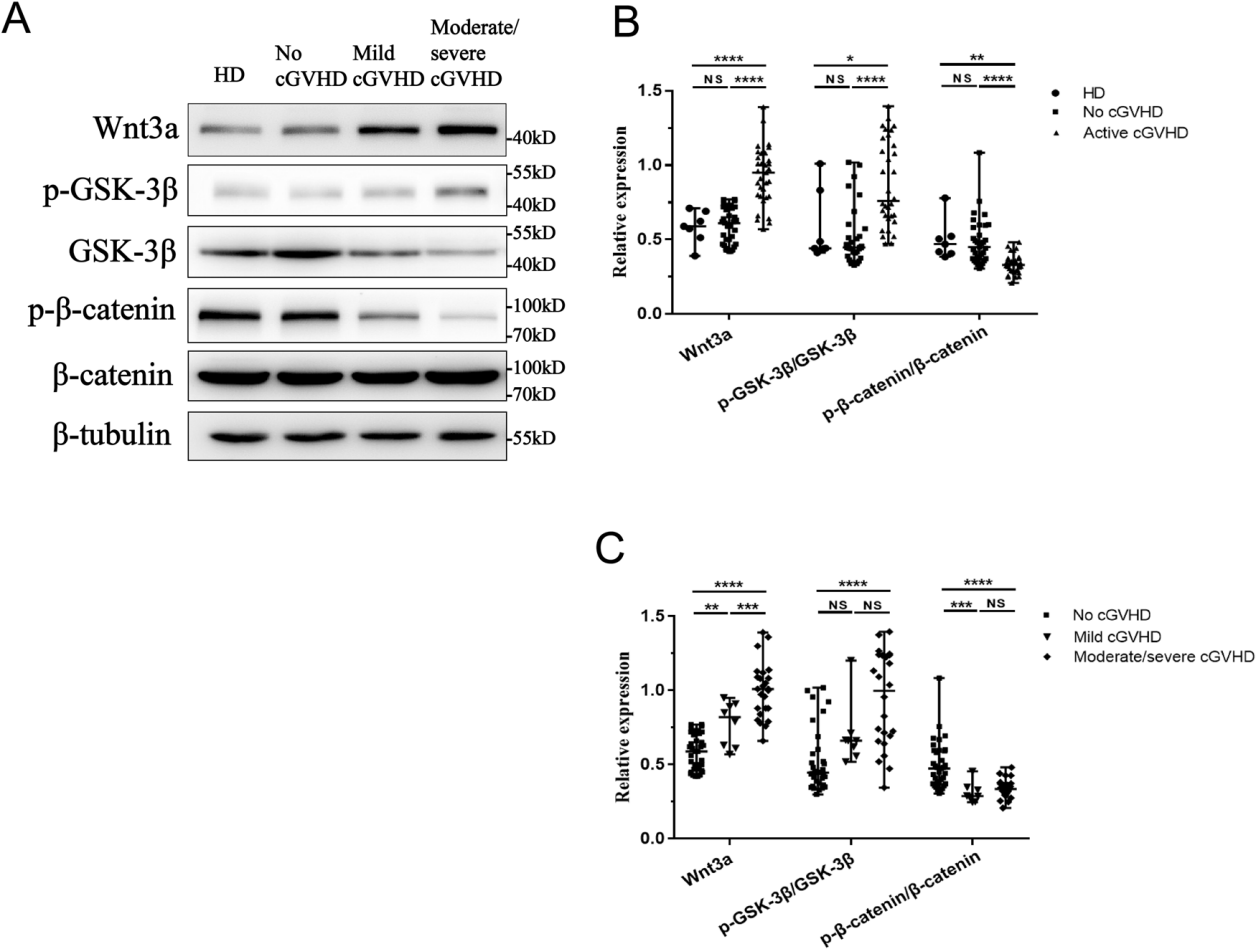
Upregulation of the Wnt/β-catenin pathway in MSCs from patients with active cGVHD. **A** Representative protein expression of Wnt3a, phosphor-GSK-3β, total-GSK-3β, phosphor-β-catenin, total-β-catenin, and β-tubulin in MSCs derived from healthy donors, patients without cGVHD, patients with mild-cGVHD and patients with moderate/severe cGVHD. **B** Relative protein expression of HD-MSCs, no cGVHD-MSCs, and active cGVHD-MSCs. **C** Relative protein expression of MSCs with different severities of cGVHD.Protein expression was normalized within each sample to the internal reference protein β-tubulin. HD-MSCs (*n* = 7), no cGVHD-MSCs (*n* = 33), mild cGVHD-MSCs (*n* = 8), and moderate/severe cGVHD-MSCs (*n* = 25). Black bars in each figure represent the median ± range. NS, not significant. *P < 0.05, **P < 0.01, ***P < 0.001, and ****P < 0.0001.

To determine whether the Wnt/β-catenin pathway mediates the abnormal differentiation of BM-MSCs, we detected the protein expression of the Wnt/β-catenin signaling pathway related proteins Wnt3a, GSK-3β, and β-catenin. Our results showed that the Wnt/β-catenin pathway ligand Wnt3a in active cGVHD-MSC was significantly increased compared to no cGVHD-MSCs (0.95 vs 0.59, *P* < 0.001) and HD-MSCs (0.95 vs 0.62, *P* < 0.001). The level of Wnt3a in moderate/severe cGVHD-MSCs was significantly higher than that of patients with mild cGVHD (1.01 vs 0.79, *P* < 0.001). Correspondingly, the phosphorylated/total GSK-3β was increased in active cGVHD-MSCs compared to no cGVHD-MSCs (0.89 vs 0.48, *P* < 0.001) and HD-MSCs (0.89 vs 0.43, *P* < 0.001). The rate of phosphorylated/total β-catenin was decreased in active cGVHD-MSC compared to no cGVHD-MSCs (0.33 vs 0.48, *P* < 0.001) and HD-MSCs (0.33 vs 0.46, *P* < 0.001, Fig. 4A–C).

**Fig. 5:**
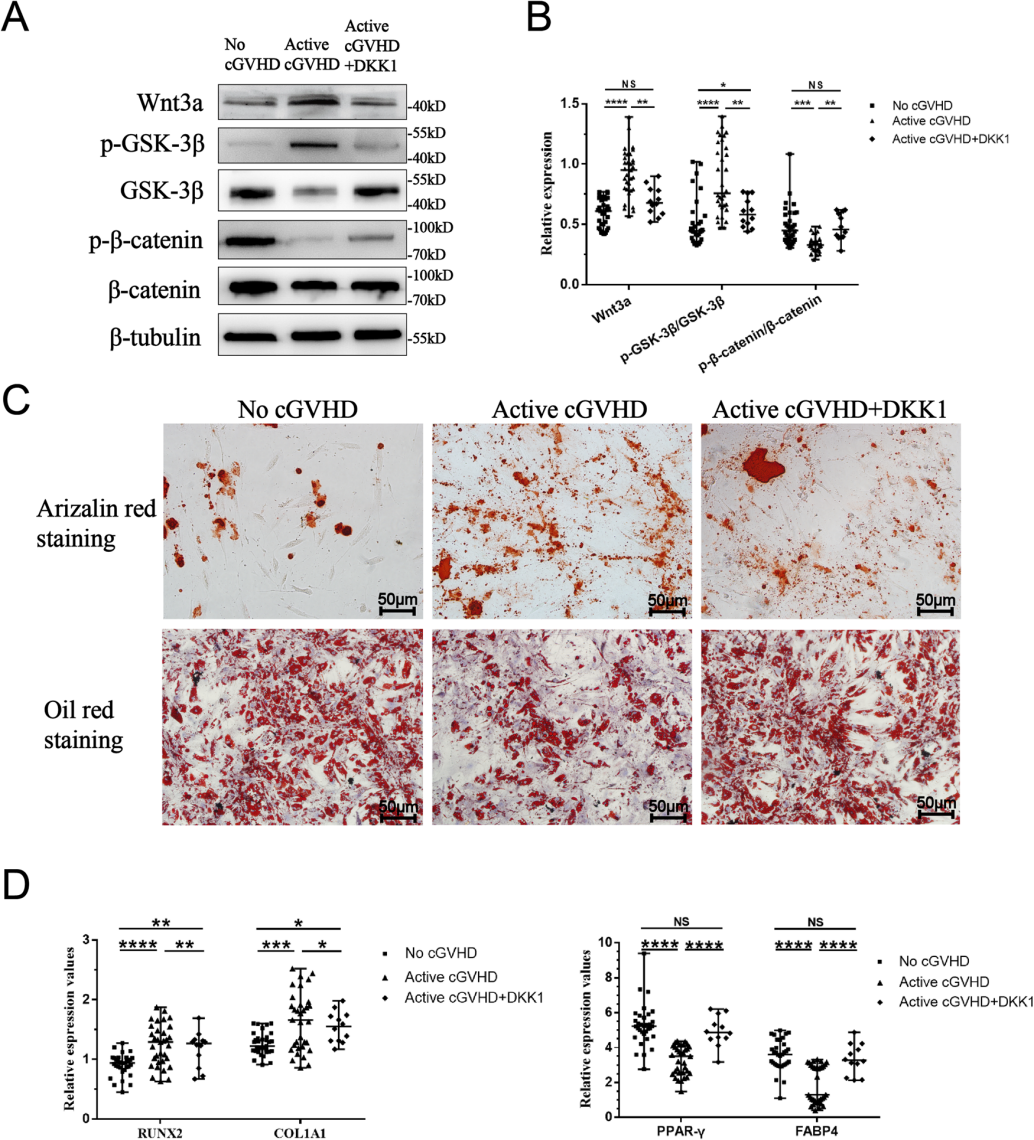
DKK1 decreased the expression of Wnt/β-catenin signaling in active cGVHD-MSCs. **A** Representative protein expression of Wnt3a, phosphor-GSK-3β, total-GSK-3β, phosphor-β-catenin, total-β-catenin, and β-tubulin in active cGVHD-MSCs treated or untreated with DKK1 (*n* = 12) and no cGVHD-MSCs (*n* = 12). **B** Relative protein expression of active cGVHD-MSCs treated or untreated with DKK1 (*n* = 12) and no cGVHD-MSCs (*n* = 12). **C** Relative gene expression of osteogenic/adipogenic marker gene. **D** Alizarin red staining, Orange-red represents calcium nodules. Oil Red O staining, red represents lipid droplets. No cGVHD-MSCs (*n* = 33), active cGVHD (*n* = 33) and DKK1 + cGVHD (*n* = 12). Black bars in each figure represent the median ± range. NS, not significant. *P < 0.05, **P < 0.01, ***P < 0.001, and ****P < 0.0001.

Since upregulation of Wnt/β-catenin signaling was found in active cGVHD-MSCs, we next evaluated the effects of inhibiting the Wnt/β-catenin pathway by DKK1 on the differentiation capacities of active cGVHD-MSCs in vitro. Our results showed that DKK1 could inhibit the levels of Wnt3a and phospho-GSK3β expression and elevate the level of phospho-β-catenin expression (Fig. 5A, B). After treated with DKK1, the calcium nodules were decreased and the lipid droplets were increased in active cGVHD-MSCs (Fig. 5C). The levels of osteogenic specific genes RUNX2 and COL1A1 were decreased after DKK1 addition (1.23 vs 1.35, *P* < 0.01 and 1.39 vs 1.66, *P* < 0.05, respectively). In contrast, the levels of adipogenic specific genes PPARγ and FABP4 were found increased in the DKK1 treated group (5.18 vs 4.50, *P* < 0.001 and 3.32 vs 1.68, *P* < 0.001, respectively).

Previous studies indicated that apart from Wnt/β-catenin pathway, there were several canonical pathways related to the osteogenic and adipogenic differentiation of MSCs, such as Akt, MAPK and Hedgehog pathway^27–29^. To further clarify whether these pathways play a role in the abnormal differentiation of active cGVHD-MSC, we then assayed the expression of proteins regulated by these pathways, Including Akt, p38 MARK and Sonic Hedgehog (SHH). The results showed that there were no significant difference in Akt, p38 MARK and SHH protein expression among BM-MSCs from HD donors, non-cGVHD patients and active cGVHD patients. And they were similar between BM-MSCs in different severities of active cGVHD patients (all *P* > 0.05, Supplemental Fig. 3A–C).

### Immunomodulation of active cGVHD-MSCs was comparable to no cGVHD-MSCs and HD-MSCs

**Fig. 6:**
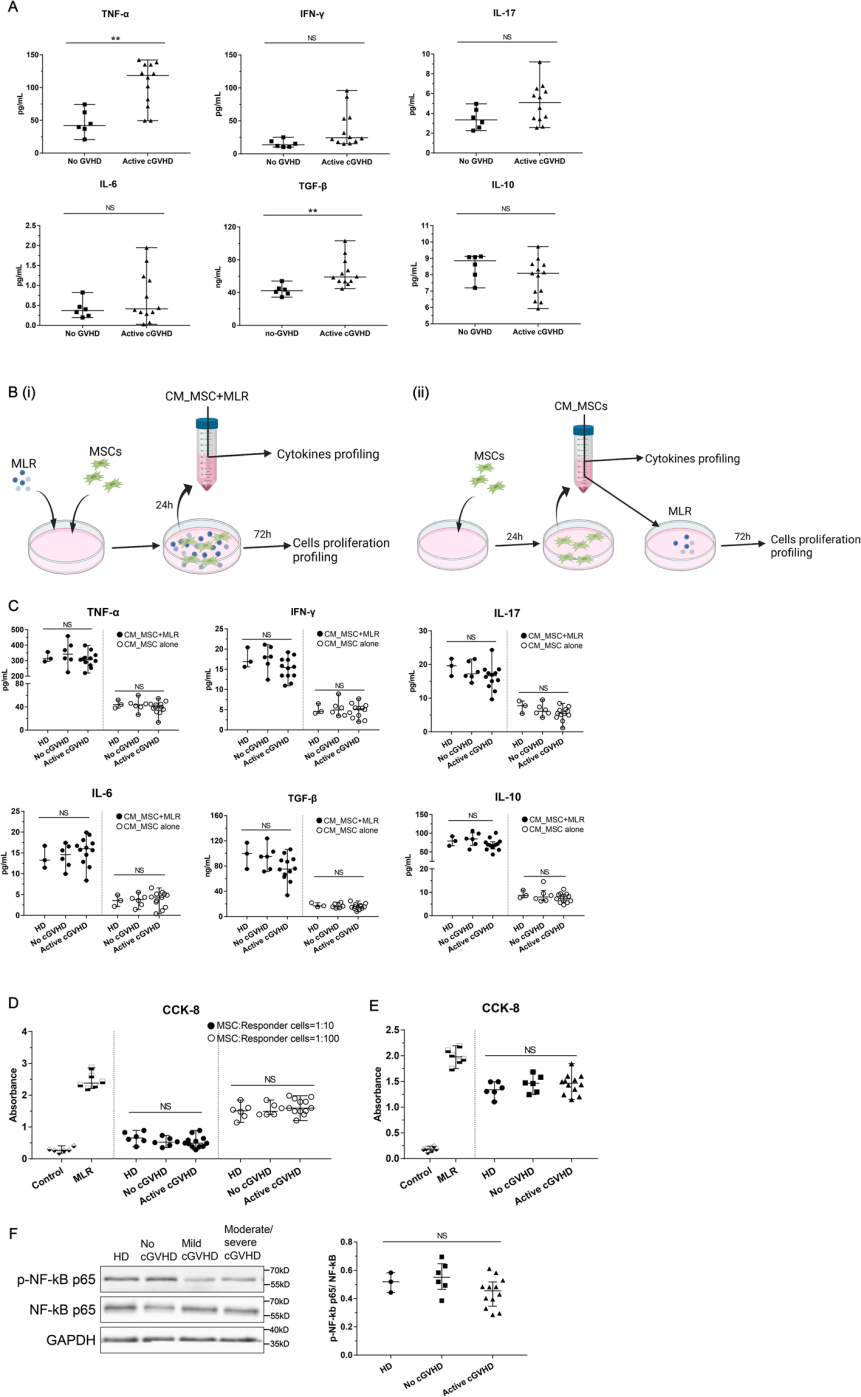
Immunomodulation of MSCs. **A** The levels of IL-6, IL-10, IL-17, TNFa, IFN-r, and TGF-b in bone marrow serum from no cGVHD and active cGVHD patients. **B** Schematic diagram showing (i) the MSC co-cultrued with MLR conditioned medium collection process and (ii) CM_MSC cultrued with MLR conditioned medium collection process. **C** The levels of IL-6, IL-10, IL-17, TNFa, IFN-r, and TGF-b in the conditioned medium of cell-cell cocultured and MSCs cultured alone. **D** The immunosuppressive function of different concentrations of cocultured MSCs from HD, no cGVHD, and active cGVHD patients. **E** The paracrine immunosuppressive function of MSCs conditioned medium with MLR. **F** Representative protein expression of NF-kB, p-NF-kB, and GAPDH in MSCs derived from healthy donors, patients without cGVHD and patients with active cGVHD. HD-MSCs (*n* = 3), no cGVHD-MSCs (*n* = 6), and active cGVHD-MSCs (*n* = 12). Black bars in each figure represent the median ± range. NS, not significant. *P < 0.05, **P < 0.01, ***P < 0.001, and ****P < 0.0001.

To further investigated the immunomodulatory function of BM-MSCs in active cGVHD patients, we evaluate the cytokine infiltration in the bone marrow serum of active cGVHD patients. The results showed that the infiltration of TNF-α and TGF-β was significantly higher in active cGVHD cohort compared to those in no cGVHD cohort (105.4 vs 46.46, pg/ml, *P* < 0.01 and 64.68 vs 42.80, ng/ml, *P* < 0.01, respectively, Fig. 6A). The levels of IFN-γ, IL-17, and IL-6 in active cGVHD cohort showed increasing trends compared to no cGVHD cohort, but did not reach statistical significance (all *P* > 0.05, Fig. 6A). These increases were related to the severities of cGVHD, the infiltration of IL-6, IL-17, TNF-α, IFN-γ, and TGF-β was significantly higher in the bone marrow serum of moderated/severe cGVHD patients compared to no cGVHD patients (IL6: 1.09 vs 0.47, pg/ml, *P* < 0.05; TNF-α: 142.00 vs 44.47, pg/ml, *P* < 0.01; IFN-γ: 55.36 vs 17.12, pg/ml, *P* < 0.001; IL17:6.19 vs 3.28, pg/ml, *P* < 0.01; TGF-β:72.72 vs 42.32, ng/ml, *P* < 0.001; Supplemental Fig. 4A). Previous studies indicated that the immunomodulation of MSCs was affected through cell-cell contact and/or cytokines secretion. Our results showed that whether in cell–cell cocultured or MSCs paracrine cultured (Fig. 6B), the levels of IL-6, IL-10, IL-17, TGF-β1, IFNγ, and TNFα in culture medium were similar among HD-MSCs, non-cGVHD MSCs and active cGVHD MSCs (all *p* > 0.05, Fig. 6C). And the capacities of inhibit mixed lymphocyte response (MLR) were similar among three groups (all *p* > 0.05, Fig. 6D, E). Specific to different severities of cGVHD, the level of IL-10 was higher in no cGVHD group compared to morderate/severe cGVHD group when cell-cell coculture with MLR (83.99 vs 63.75, pg/ml, *P* < 0.05, Supplemental Fig. 4A). The other cytokines and the inhibition of MLR were similar in different severities of cGVHD-MSCs (all *p* > 0.05, Supplemental Fig. 4A–C). It has been documented that NF-κB activation mediates the cytokine factors secretion by MSCs. We then assay the activation of NFkB. The results showed that the levels of p-NFkB/NFkB in BM-MSCs showed no difference among three groups (*P* > 0.05, Fig. 6F and Supplemental Fig. E).

## Discussion

Our current study demonstrates that the osteogenic and adipogenic capacities of BM-MSCs were impacted in the patients with active cGVHD. Upregulation of the Wnt/β-catenin pathway participates in the dysfunction of MSCs in the patients with active cGVHD.

A growing number of studies from animal and human has demonstrated that the dysfunctions of MSCs are associated with autoimmune diseases. Recently, Ding et al. reported that the numbers of MSCs were decreased in the early period after allo-HSCT, then slowly increased over time following allo-HSCT^30^. It has also been reported that the self-renew and differentiation capacities were decreased in the patients with aGVHD compared with the patients without GVHD^23^. Wang et al. recently reported that the morphology, immunophenotype, self-renewal, and differentiation capacity of BM-MSCs were similar between the patients with cGVHD and those without cGVHD^31^. In this study, we found the frequencies of MSCs in the patients whether with or without cGHVD after allo-HSCT were decreased compared to heathy donors, while were similar between the patients with active cGVHD and the patients without cGVHD.

Immunomodulation is a key function of MSC through paracrine or/and cell-cell contact in HSCT^32^. Our study revealed the severity of inflammatory infiltration was significantly enhanced in the bone marrow serum of moderate/severe cGVHD patients compared to that of no cGVHD patients. The NF-κB signaling pathway plays a critical role in the expression of cytokines and regulates immune responses. It has been reported that NF-κB activation mediates cytokine factor secretion by MSCs^33,34^. In this study, we found that the immune regulation function of BM-MSCs did not affect through the activation of NF-κB by the inflammatory infiltration in bone marrow serum. The paracrine and immunosuppressive abilities showed no difference between active cGVHD-MSCs, no cGVHD-MSCs, and HD-MSCs, no matter MSCs were cultured alone or cocultured with MLR.

With respect to the differentiation functions of MSCs, we found the adipogenic capacity of MSCs was markedly decreased in the patients with active cGVHD, while osteogenic differentiation capacity was increased in the patients with active cGVHD compared with those without cGVHD. Moreover, we further demonstrated that these dysfunctions of MSCs were correlated with the clinical grade of cGVHD. Our findings were inconsistent with Wang’s study^31^, in which the reason might be that: (1) The time after transplantation in our patients was shorter than Wang’s study (median, 9.5months vs20 months). (2) In our study, the history of II-IV aGVHD was similar between active cGVHD patients and no cGVHD patients (54.5% vs 57.6%, *P* = 0.80), However, there is a higher incidence of II-IV aGVHD history in cGVHD cohort compared to no cGVHD cohort (44.0% vs 12.5%, *P* = 0.034) in Wang’s study.

Wnt/β-catenin signaling is an evolutionarily conserved intracellular signaling and plays an important role in the osteogenic and adipogenic differentiation of MSCs^35,36^. Activation of Wnt/β-catenin signaling is related to the decrease of osteogenic differentiation and the increase of adipogenic differentiation^37,38^. Briefly, the increased of Wnt3a upregulated the phosphorylation of GSK3β, and p-GSK3β subsequently resulted in nuclear β-catenin accumulation, induced the promotion of osteogenic and inhibition of adipogenic. The role of Wnt/β-catenin signaling in the pathogenesis of autoimmune diseases is gaining more and more attention. Nakamura et al. reported that the Wnt/β-catenin ligands were significantly upregulated in RA synovium^39^. Briolay et al. reported that in patients with ankylosing spondylitis, Wnt/β-catenin signaling activation might be involved in the effects of inflammation on bone formation by promoted MSCs osteogenic differentiation^25^. Wang et al. revealed an elevated Wnt/β-catenin signaling activity in human renal tissues of patients with lupus nephritis(LN), suggesting that a dysregulated Wnt/β-catenin signaling might play a role in the renal fibrosis^40^. Recently, Chen et al. reported that Wnt/β-catenin signaling pathway was implicated in the activation of fibrogenic program in fibroblasts in cGVHD mice model^41^. In this study, we found that the Wnt/β-catenin signaling pathway ligand Wnt3a was significantly overexpressed in BM-MSCs from the patients with active cGVHD, leading to the decreased of the phosphor-β-catenin and the dysfunction of osteogenesis and adipogenesis. We further demonstrated that the overexpression of Wnt3a was associated with the severity of cGVHD. With the block of Wnt3a by DKK1, the dysfunction of osteogenesis and adipogenesis in active cGVHD MSCs were restored. Based on these results, we demonstrated that upregulation of Wnt3a and subsequently downstream protein abnormal through Wnt/β-catinin signaling pathway participates in the dysfunction of MSCs in patients with active cGVHD. A potential mechanism leading to the dysregulation of Wnt/β-catinin signaling pathway might be the infiltration of proinflammatory cytokines, because growing evidence showed that the overexpression of Wnt ligand induced by proinflammatory cytokines contributes to the pathogenesis of autoimmune diseases^25,42,43^. In this study, we found the proinflammatory cytokines, such as IL-6, IL-17, IFN-γ, and TNF-α, were significantly increased in the bone marrow serum. But the relationship between inflammatory cytokines and Wnt/β-catinin signaling pathway in the bone marrow of cGVHD patients remains unclear, and the underlying mechanism leading to the dysregulation of the Wnt/β-catinin pathway in cGVHD-MSCs needs further explored. Our findings indicated that the differentiation of BM-MSCs in active cGVHD patients were abnormal through the Wnt/β-catinin pathway, these abnormalities may contribute to the development of cGVHD. DKK1, the inhibitor of the Wnt pathway, can reverse these abnormalities, thus presenting a novel therapeutic target for cGVHD. Furthermore, the abnormality of BM-MSCs may become one of the indicators for predicting cGVHD and evaluating the severity of cGVHD.

## Conclusion

In conclusion, the adipogenic and osteogenic capabilities of BM-MSCs were abnormal in the patients with active cGVHD. The dysfunctions of BM-MSCs were mediated by the upregulated of Wnt3a through Wnt/β-catenin signaling pathway.

## Material and methods

### Study design and patient eligibility

The study included 33 patients who were older than 14 years old and developed primary cGVHD after hematopoietic stem cell transplantation at Nanfang Hospital. Thirty-three patients without cGVHD were closely matched to the cGVHD cohort according to time after transplantation, age, gender, stem cell source, conditioning regimen, GVHD prophylaxis and grade of acute GVHD. An additional 7 healthy stem cell transplant donors were recruited for this study. Fresh bone marrow samples were collected from the outpatient bone marrow aspiration. Diagnosis of cGVHD and its severity were established at the time of sample collection using recent National Institutes of Health guidelines^44^. Patients with active cGVHD were defined as requiring prednisone or multiagent immunosuppression^45^. Patients with no cGVHD were defined as those who never developed cGVHD after HSCT. Exclusion criteria were as follows: (1) Patients with inactive cGVHD, defined as a complete response to immune-suppressive therapy for active cGVHD; (2) Patients with recurrent, persistent, or late onset acute GVHD, defined as features of classic acute GVHD occurring beyond 100 days post-transplantation or DLI. This study was conducted in accordance with the Declaration of Helsinki and approved by the institutional review board of Nanfang Hospital (Ethical approval No. NCT04692376). All patients and healthy donors gave written informed consent to participate in the study.

### Transplants

HLA-haploidentical donor (HID) patients were transplanted with a combination of bone marrow (BM) and peripheral blood stem cell (PBSC) grafts, whereas HLA-matched sibling donor (MSD) patients and HLA-matched unrelated donor (MUD) patients received PBSC grafts^46^. Cyclosporine A (CsA), methotrexate (MTX), and mycophenolate mofetil (MMF) were administered to patients undergoing MSD transplant for GVHD prophylaxis. CsA, MTX, MMF and antithymocyte globulin (ATG) were administered to patients undergoing HID transplants for GVHD prophylaxis, and CsA, MTX, and ATG were administered to patients undergoing MUD transplants^46^. aGVHD and cGVHD treatments were detailed in previous report^47^.

### Isolation, culture and identification of MSCs

Isolation, culture, and identification of human MSCs were as described in our previous publication^18^. Briefly, BM samples were collected from participants, and mononuclear cells (BMMNCs)were isolated by Ficoll gradients (Ficoll-PaqueTBD Science, Tianjin, China). The cells were cultured with Dulbecco’s modified eagle medium nutrient mixture F-12 (Gibco, San Diego, CA) containing 10% fetal bovine serum (Gibco, New York, US). When cells reached 80–90% confluence, they were trypsinized by trypsin-EDTA (Gibco, San Diego, CA) and designated as passage 1. These cells were further passaged at a ratio of 1:3. Passages 2 and 3 MSCs were used for this study. MSCs were identified by antigen expression with flow cytometry as well as by their adipogenic and osteogenic differentiation capacities^18^.

### MSCs frequency in BMMNCs

The MSCs frequency was defined as the number of colony-forming unit fibroblasts (CFU-F) per 10^6^ BMMNCs^48^. Briefly, the BMMNCs were seeded in 6-well plates at a density of 1 × 10^5^ cells/well, cultured for 7 days, and then the numbers of colonies were calculated.

### Flow cytometry analysis

The antibodies and reagents used for flow cytometry analysis were as follows: PE-CD19 (HIB19), APC-CD29 (TS2/16), PE-CD31 (PECAM-1), PE-CD34 (QBEND/10), PERCP5.5-CD44 (IM7), FITC-CD45 (HI30), PECY7-CD90 (Thy-1) and PE-CD105 (SN6) were purchased from ThermoFisher Scientific (San Diego, CA). All staining was performed according to the manufacturer’s instructions. Flow cytometric analysis was performed on a FACS Canto™ II Flow Cytometer (BD Biosciences, San Diego, CA), and the resulting data were analyzed with FlowJo software (Tree Star, Ashland, OR).

### MSCs proliferation and apoptosis assay

MSCs proliferation was measured using the Cell Counting Kit-8 according to the manufacturer’s protocol (Yeasen, Shanghai, China). An annexin V-fluorescein isothiocyanate (FITC)/propidium iodide (PI) cell apoptosis kit (Yeasen, Shanghai, China) was used to quantify cell apoptosis. The percentages of early apoptotic cells (annexin V+/PI−) and late apoptotic cells(annexin V+/PI+) were analyzed using FACSCanto™ II flow cytometry (BD Biosciences, San Diego, CA).

### MSCs differentiation assays

For osteogenic and adipogenic differentiation of MSCs in vitro, 60%–70% confluent MSCs were cultured in osteogenic or adipogenic medium (Cyagen Biosciences, Santa Clara, CA)for differentiation. Alizarin red staining was used to evaluate calcium deposition while Oil Red O was used to visualize lipid vacuoles.

### RNA isolation and real-time quantitative polymerase chain reaction (RT-qPCR) analysis

RNA was isolated from cells using RNAiso Plus (Takara Bio Inc., Shiga, Japan) and complementary DNA (cDNA) was prepared using a Hifart II First Strand cDNA Synthesis Kit (Yeasen, Shanghai, China) according to the manufacturer’s instructions. The cDNA thus obtained was subjected to real-time quantitative PCR with the Hieff qPCR SYBR Green Master Mix kit (Yeasen, Shanghai, China) using the primers. Each sample was analyzed in triplicate and all results were normalized to the expression of glyceraldehyde 3-phosphate dehydrogenase (GAPDH). The primers used are as follows:

PPAR*γ*, forward: 5′-AGCCTGCGAAAGCCTTTTGGTG-3′,

reverse: 5′-GGCTTCACATTCAGCAAACCTGG-3′.

Runx2, forward: 5′-GCAGTTCCCAAGCATTTCAT-3′,

reverse: 5′-CTGGCGGGGTGTAAGTAAAG-3′.

FABP4, forward: 5′-GCAGAAATGGGATGGAAAATC-3′,

reverse: 5′-CTCATAAACTCTCGTGGAAGTGA-3′.

Col1a1, forward: 5′-CTGCTGGACGTCCTGGTGAA-3′,

reverse: 5′-ACGCTGTCCAGCAATACCTTGAG-3′.

GAPDH, forward 5′-AGAAGGCTGGGGCTCATTTG-3′,

reverse 5′-AGGGGCCATCCACAGTCTTC-3′.

### Western blot analysis

Western blot analyses were conducted on the protein of lysates from in vitro cultured MSCs. In the Dickkopf related protein 1 (DKK1) groups, 100 ng/mL DKK1 (R&D Systems, USA) was directly added to the cells and incubated for 72 h. The cells were lysed in RIPA lysis buffer containing proteinase inhibitor and PhosSTOP (Fabio Science, China). Equivalent amounts of cell lysate were separated by SDS–polyacrylamide gel electrophoresis (PAGE) and blotted on polyvinylidene fluoride membrane (Millipore Corp, Oakville, Canada). After blocking in 5% skim milk, the membranes were incubated with primary antibodies and corresponding secondary antibodies.β-Tubulin was used as an endogenous control to normalize protein expression level. Specific antibodies were incubated as follows: GSK-3β (D5C5Z), Phospho-GSK-3β (Ser9), β-catenin (D10A8), phospho-β-catenin (Thr41), Akt (C67E7), p-Akt (Ser473), p38 MAPK (D13E1), p-p38 MAPK (Thr180/Tyr182), NF-kB p65 (D14E12), p-NF-kB p65 (Ser536) and Wnt3a(C64F2) (Cell Signaling Technology, Danvers, MA), GAPDH (FD0063) and β-tubulin (FD0064) (Fude Biological Technology, Hangzhou, China); and horseradish peroxidase-linked secondary antibody (Abcam, Cambridge, UK). Detection was carried out with ECL kits (Millipore Corp, Oakville, Canada) according to the manufacturer’s instructions. Images were obtained using a FluorChem HD2 System (Tanon, Shanghai, China).

### Enzyme-linked immunosorbent assay (ELISA)

Bone marrow serum, supernatants from cultures of MSCs or cocultures of MSCs/PBMCs were collected and cytokines were measured using commercially available enzyme-linked immunosorbent assay ELISA kits: IL-6 (70-EK106), IL-10 (70-EK110), IL17 (70-EK117), TNF-α (70-EK182), IFN-γ (70-EK180) and TGF-β (70-EK981; MultiSciences, Hangzhou, China) according to the manufacturer’s instructions.

### Mixed lymphocyte reaction

To determine whether MSCs regulate the immune response, MSCs in gradient concentrations (5 × 104/well; 0.5 × 104/well) were seeded in a 96-well round-bottom plate and cultured at 37 °C in a humidified incubator for 4 h to allow cell adherence. Stimulator cells were PBMCs irradiated with 3,000 rads. The stimulator cells were mixed with responder cells at a ratio of 1:10 in MLR culture medium, and then added to the MSCs pre-seeded plates or the concentrated conditioned medium collected from MSCs. The ratio of MSCs to responder cells was 1:10 and 1:100. After 72 h of coculture, cell proliferation was quantified using a CCK-8 according to the manufacturer’s instructions.

### Statistical analysis

A descriptive analysis of all variables was performed, including median, range, minimum and maximum values for continuous variables and numbers and frequencies for categorical variables. For categorical variables, the chi-square statistic or Fisher exact test was used to establish differences in their distribution; the Wilcoxon rank-sum test was used to compare continuous variables. Comparisons of three groups were analyzed using one-way ANOVA test. Comparison of two groups was analyzed using an unpaired two-tailed Student’s *t* test. All data were analyzed with GraphPad Software (Prism Version 6.0; GraphPad Software, San Diego, CA) and SPSS version 22.0 (SPSS, Chicago, USA). Statistical significance was defined as *P* value < 0.05.

## Supplementary information

Supplemental figure legend

Supplemental Figure 1

Supplemental Figure 2

Supplemental Figure 3

Supplemental Figure 4
