# Supplementary material for: Wnt/β-catenin signaling mediates the abnormal osteogenic and adipogenic capabilities of bone marrow mesenchymal stem cells from chronic graft-versus-host disease patients
Source: Cell Death Dis. 2021 Mar 23;12(4):308. doi: 10.1038/s41419-021-03570-6 (PMC7988169; doi:10.1038/s41419-021-03570-6)
Supplement: Supplementary file 1 — Supplemental figure legend [file 41419_2021_3570_MOESM1_ESM.docx]

**Supplemental Figure 1. Similar proliferation of MSCs in three groups, measured by CCK-8.**

(A) The proliferation of HD-MSCs, no cGVHD-MSCs and active cGVHD-MSCs were measured by CCK-8. (B) The proliferation of MSCs with different severity of cGVHD. HD-MSCs (n=7), no cGVHD-MSCs (n=33), and active cGVHD-MSCs (n=33). Black bars in each figure represent the median ± range. NS, not significant.

**Supplemental Figure 2. Similar results among moderate cGVHD-MSCs and severe cGVHD-MSCs.**

(A) The frequencies of MSCs from moderate cGVHD and severe cGVHD. (B) Passage time of moderate cGVHD-MSCs and severe cGVHD-MSCs from P1-P4. (C) Representative apoptosis analysis in MSCs by flow cytometry. The percentage of early and late apoptotic cells in morderate cGVHD-MSCs and severe cGVHD-MSCs were quantified are shown in histograms. (D) Gene expression of osteogenic markers RUNX2, COL1A1 and gene expression of adipogenic markers PPAR-γ and FABP4 in morderate cGVHD-MSCs and severe cGVHD-MSCs. (E) Representative protein expression of Wnt3a, phosphor-GSK-3β, total-GSK-3β, phosphor-β-catenin, total-β-catenin and β-tubulin in MSCs derived from moderate and severe cGVHD. HD-MSCs (n=3), no cGVHD-MSCs (n=6), and active cGVHD-MSCs (n=12). Black bars in each figure represent the median ± range. *P<0.05, NS, not significant.

**Supplemental Figure 3. Expression of differentiation related proteins in MSCs.**

**(A)** Representative protein expression of Alt, p-Akt, p38 MAPK, p-p38 MAPK, Shh and GAPDH in MSCs derived from healthy donors, patients without cGVHD, patients with mild-cGVHD and patients with moderate/severe cGVHD. (B) Relative protein expression of HD-MSCs, no cGVHD-MSCs and active cGVHD-MSCs. (C) Relative protein expression of MSCs with different severities of cGVHD.Protein expression was normalized within each sample to the internal reference protein GAPDH. HD-MSCs (n=3), no cGVHD-MSCs (n=6), mild cGVHD-MSCs (n=6) and morderate/severe cGVHD-MSCs (n=6). Black bars in each figure represent the median ± range. NS, not significant.

**Supplemental Figure 4. Immunomodulation of MSCs.**

(A)The levels of IL-6, IL-10, IL-17, TNFa, IFN-r and TGF-b in bone marrow serum from different severities of cGVHD. (B) The levels of IL-6, IL-10, IL-17, TNFa, IFN-r and TGF-b in the conditioned medium of cell-cell co-cultured and MSCs cultured alone from different severities of cGVHD. (C) The immunosuppressive function of different concentrations of MSCs from different severities of cGVHD-MSCs. (D) The immunosuppressive function of different severities of cGVHD-MSCs conditioned medium with MLR. (E) Representative protein expression of NF-kB, p-NF-kB and GAPDH in MSCs derived from different severities of cGVHD. No cGVHD-MSCs (n=6), mild cGVHD-MSCs (n=6) and morderate/severe cGVHD-MSCs (n=6). Black bars in each figure represent the median ± range. NS, not significant. *P<0.05, **P<0.01, ***P<0.001 and ****P<0.0001.
