# Supplementary figures and images for: Wnt/β-catenin signaling mediates the abnormal osteogenic and adipogenic capabilities of bone marrow mesenchymal stem cells from chronic graft-versus-host disease patients
Source: Cell Death Dis. 2021 Mar 23;12(4):308. doi: 10.1038/s41419-021-03570-6 (PMC7988169; doi:10.1038/s41419-021-03570-6)

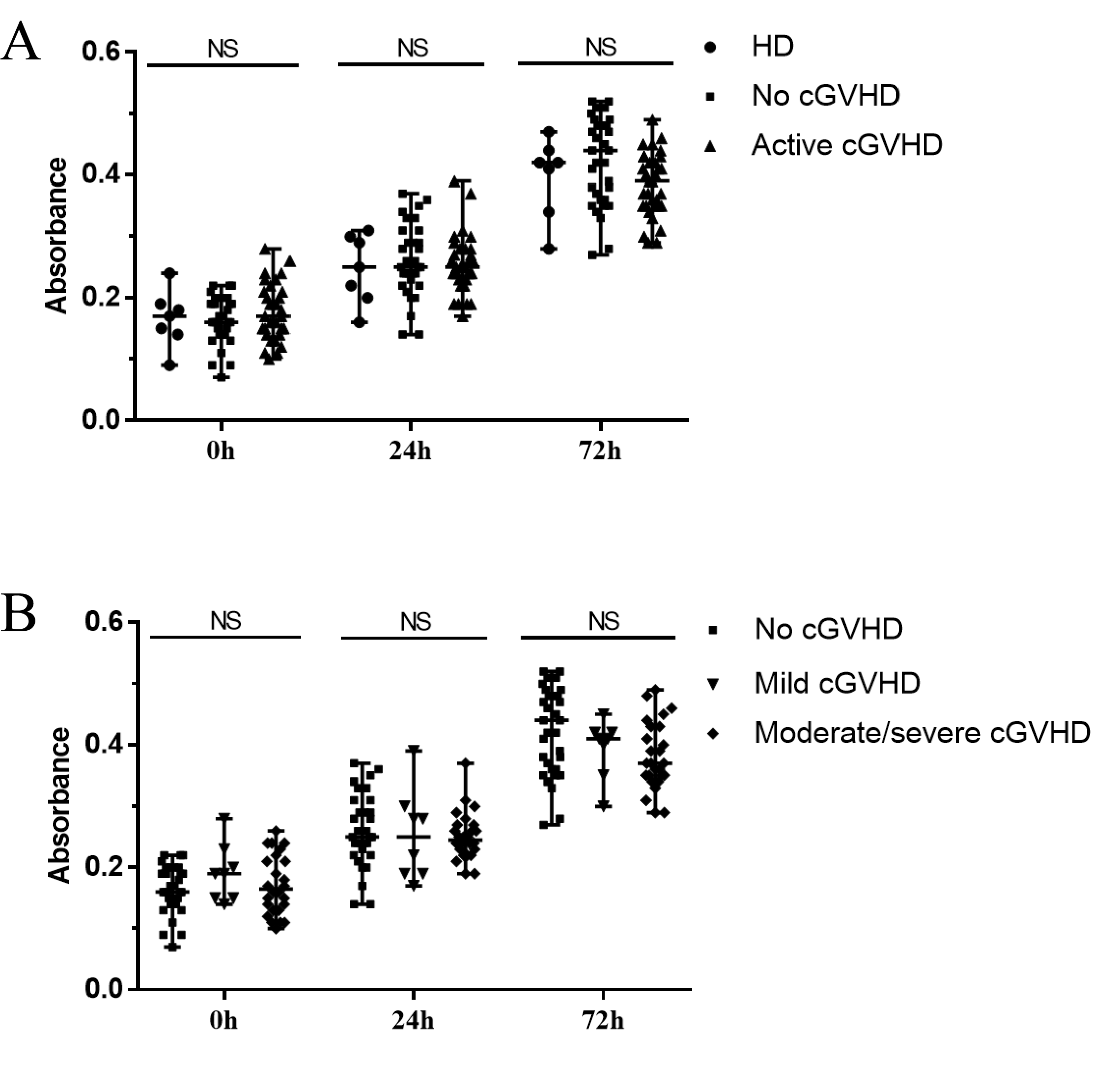

Supplement: Supplementary file 2 — Supplemental Figure 1 [file 41419_2021_3570_MOESM2_ESM.png]

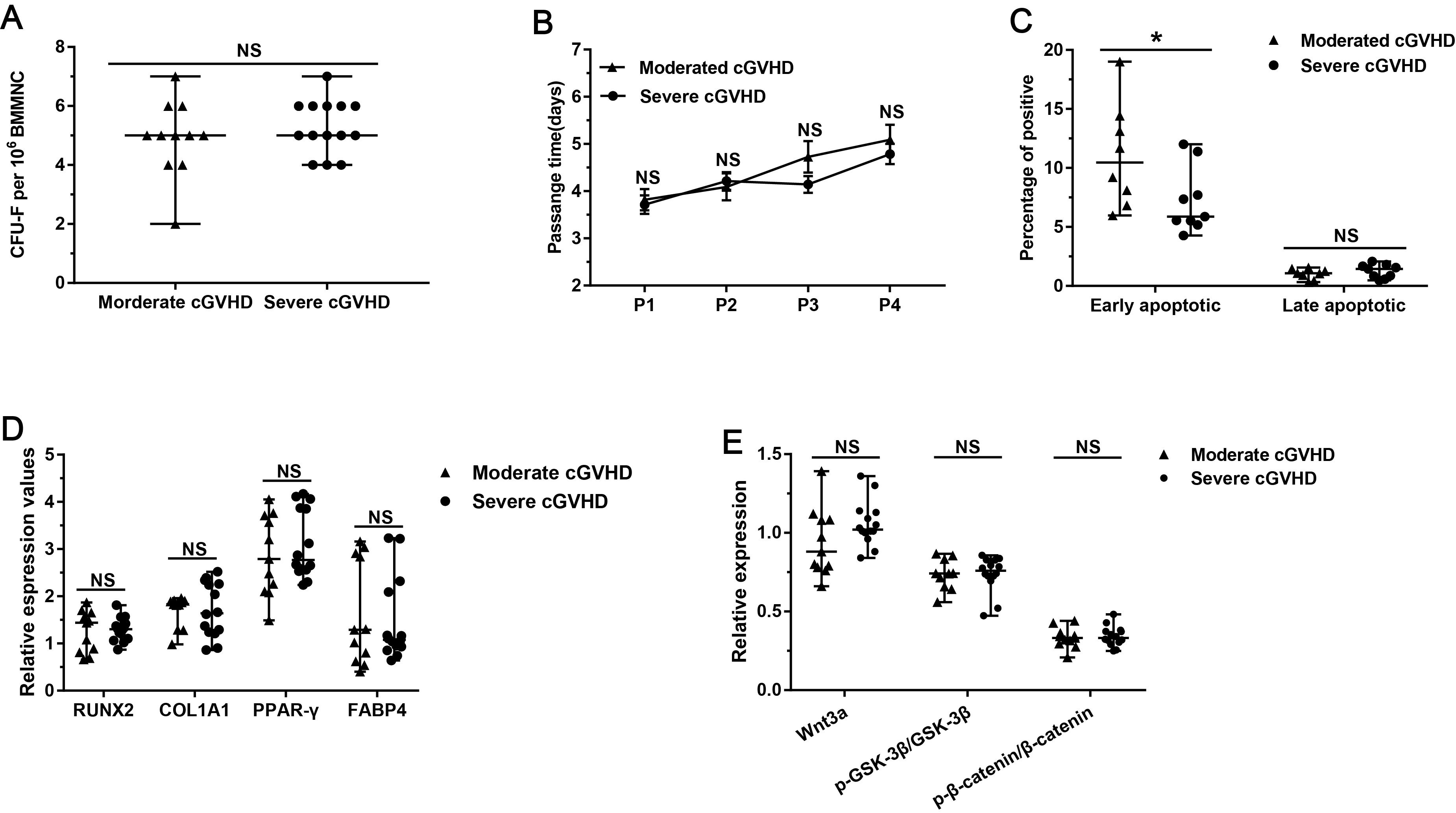

Supplement: Supplementary file 3 — Supplemental Figure 2 [file 41419_2021_3570_MOESM3_ESM.png]

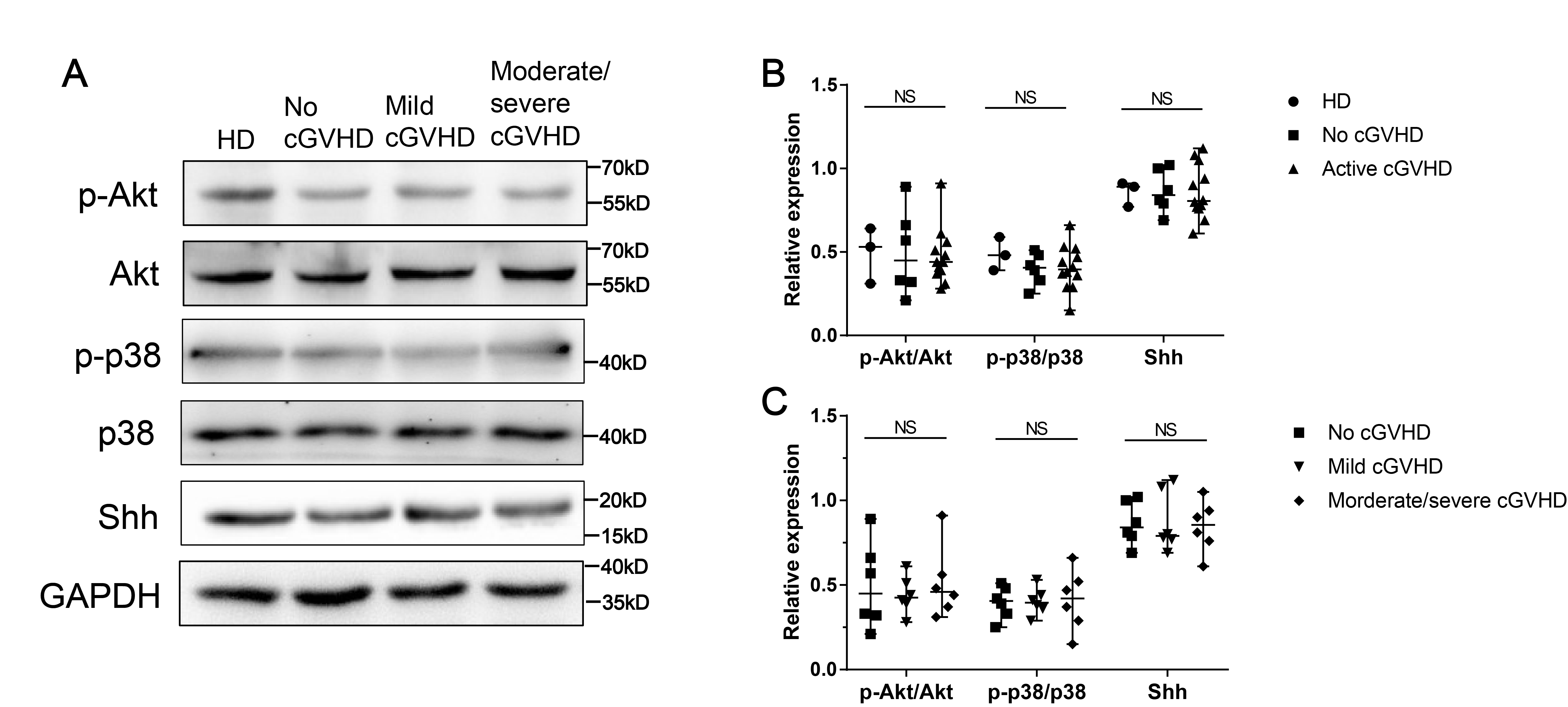

Supplement: Supplementary file 4 — Supplemental Figure 3 [file 41419_2021_3570_MOESM4_ESM.png]

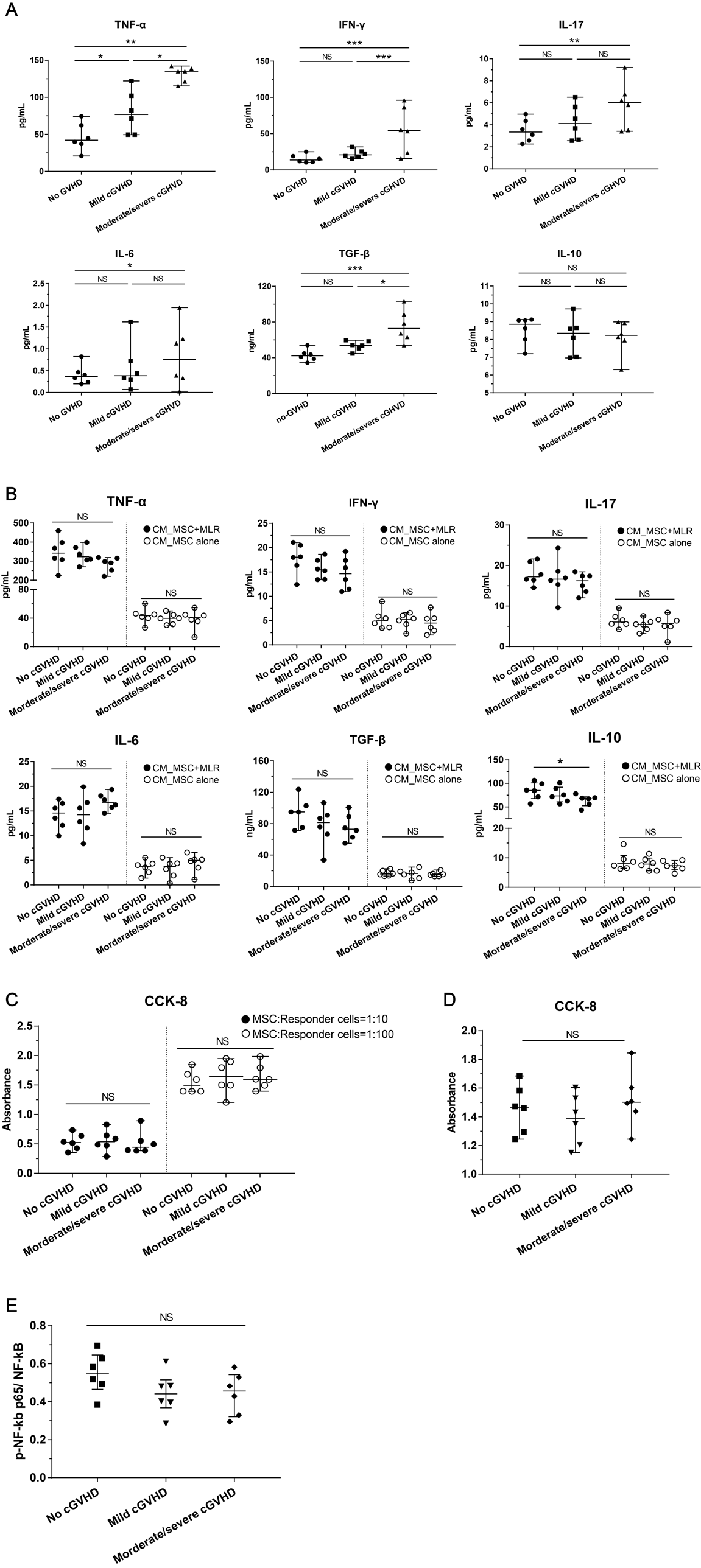

Supplement: Supplementary file 5 — Supplemental Figure 4 [file 41419_2021_3570_MOESM5_ESM.png]
